# Supplementary material for: Scalable neighbour search and alignment with uvaia
Source: PeerJ. 2024 Mar 6;12:e16890. doi: 10.7717/peerj.16890 (PMC10924453; doi:10.7717/peerj.16890)
Supplement: Supplemental Information 1 [file peerj-12-16890-s001.pdf]

**Title:** Scalable neighbour search and alignment with uvaia
**Authors:** Leonardo de Oliveira Martins[1,*], Alison E. Mather[1,2], Andrew J. Page[1]
**Affiliations:**
[1] Quadram Institute Bioscience, Norwich, UK
[2] University of East Anglia, Norwich, UK
* Corresponding email: Leonardo.de-Oliveira-Martins@quadram.ac.uk

# Supplementary Text and Figures

## Phylogenetic analysis including partially ambiguous sites

Partially ambiguous sites are those where the identity of the nucleotide at a particular position is uncertain. This can happen for a variety of reasons, such as sequencing error, limited coverage, or the presence of populational polymorphisms. When these sites are analysed by uvaia, they can be scored as either a match or not with a corresponding site in another sequence, depending on the distance chosen. This information can then be used to calculate the overall similarity between two sequences, which can be then used to infer their evolutionary relationships.

The phylogenetic likelihood function handles naturally ambiguous sites (Felsenstein, 2003; Yang, 2014), and this principle (of partial uncertainty) has been extended in polymorphism-aware models (De Maio et al., 2013), single-cell phylogenetics (Kozlov et al., 2020), and sequencing error models (Burgess & Yang, 2008). The explicit incorporation of partially ambiguous sites as informative phylogenetic characters led to distances equivalent to the number of partial matches reported by uvaia (Joly et al., 2015; Potts et al., 2014). These distances fare well in comparison to others (Zhao et al., 2022), and have been used in phylogenetic studies (Scheunert & Heubl, 2017).

To compare sequences selected by uvaia with SNP-based neighbours in a phylogenetic context, we used the "number of partial mismatches" from uvaia along with a set of neighbouring sequences estimated with UShER (Turakhia et al., 2021). For this comparison, we employed a small sequence database and selected a query sequence at random. Specifically, we utilised the same 1,000 sequences as before and created a NJ tree using rapidnj. This tree was then further optimised under parsimony (Ye et al., 2022) to generate the mutation-annotated tree (MAT) object used by UShER (Turakhia et al., 2021). The sequence Wuhan-Hu-1 was used as reference. We randomly selected a sequence from this set, England/NORW-316F2DC/2022, and used uvaia to find all sequences with zero partial mismatches (sequences where all differences are due to gaps/indels, ambiguous or partially ambiguous sites). In total 27 such sequences were found. At the same time we extracted the 40 nearest samples to it according to the MAT (McBroome et al. 2021), which we will refer to as the UShER neighbours. The objective here is to see if the methods are equivalent, and if including partially ambiguous sites as uvaia does makes any difference phylogenetically.

From all neighbours found by any of the methods, we obtained 12 sequences uniquely by uvaia, 25 sequences which were found only by UShER, and 15 sequences were found as neighbours by both methods. In Figure S1 we have a maximum likelihood tree for these 53 sequences under an HKY model (Hasegawa et al., 1985) with rate heterogeneity between sites modelled through a Gamma distribution (Yang, 1994), estimated with RAxML-NG (Kozlov et al., 2019). We can see that some sequences found by uvaia but not by UShER are inferred as being phylogenetically closer to the query sequence than some found by UShER.

Further investigation showed that sequences found only by uvaia do have a higher parsimony distance to the query sequence according to the MAT object (Figure S2), but which would eventually be selected given a high enough number of neighbours. Furthermore he sequences chosen only by uvaia had on average 3 partially ambiguous sites, compared to the average of 0.5 for those chosen by UShER (including those also chosen by both uvaia and UShER, data not shown).

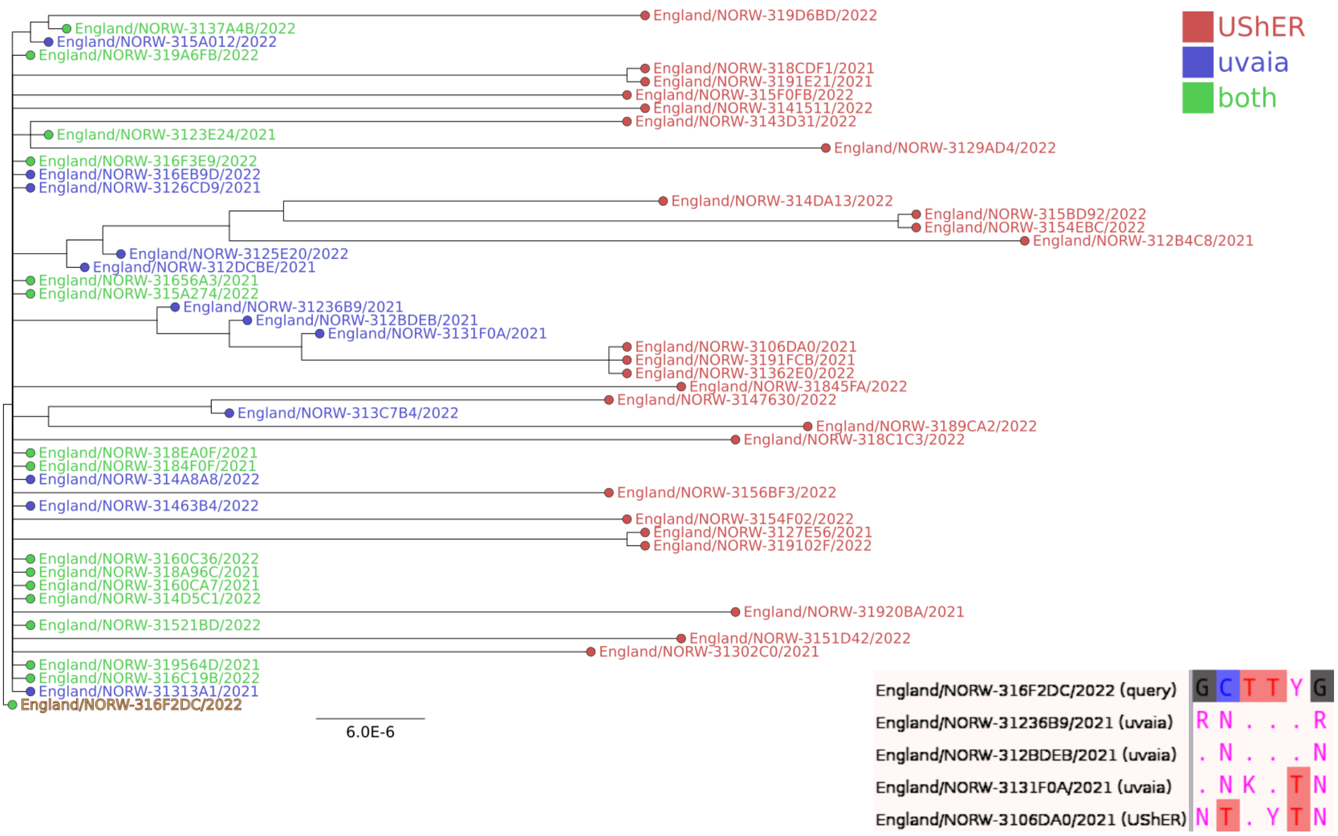


Figure S1: Maximum likelihood tree with neighbours to the query sequence England/NORW-316F2DC/2022 according to UShER (red), to uvaia (blue), and to both (green). The inset shows the sites where an arbitrary set of chosen sequences differ, to see how likelihood (and Bayesian) methods can, in principle, use partially ambiguous sites to refine the tree estimation (these sequences are not part of a polytomy). Arbitrarily short branches were replaced by a polytomy by RAxML-NG. For visualisation purposes, the tree was rooted at the query sequence.

Here we can see that (1) considering partial matches can allow us to find close neighbours which might be disconsidered otherwise, and that (2) these partially ambiguous sites can be used by existing tree inference programs, besides allowing for proper incorporation into (future) phylogenetic inference models.

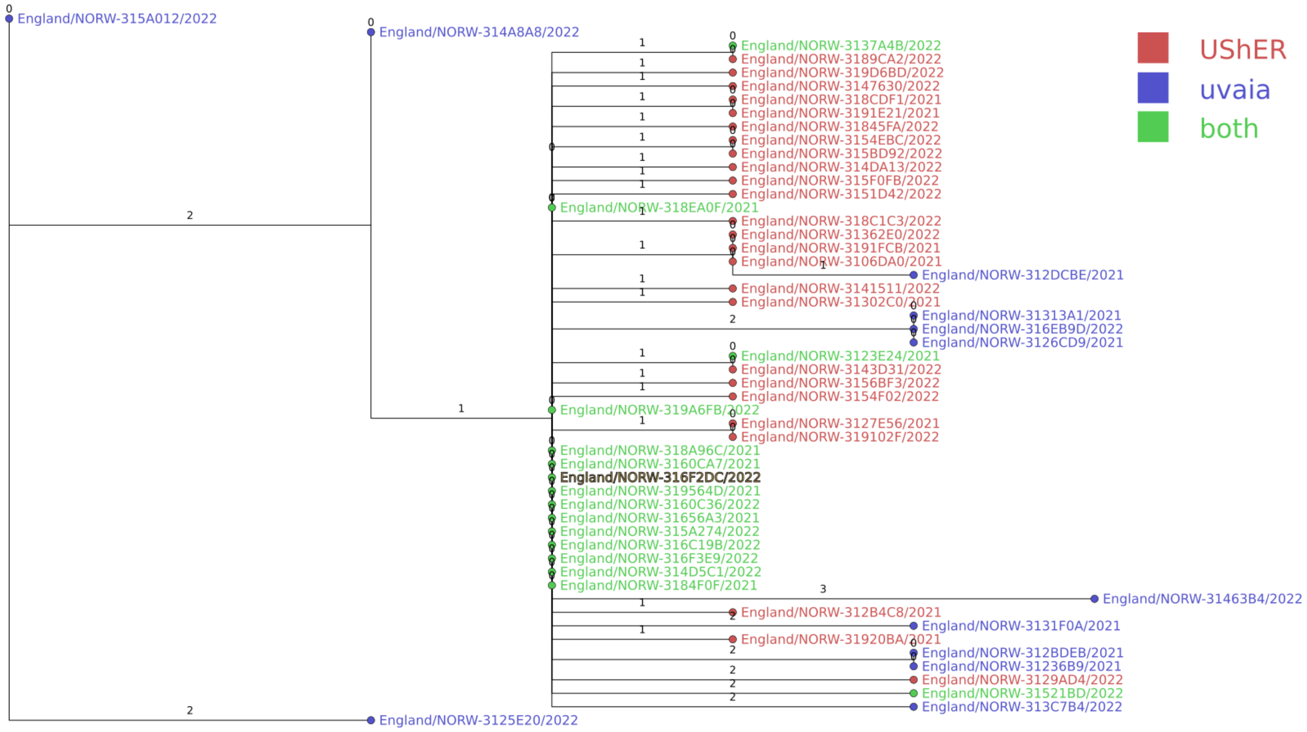

Figure S2: Subtree extracted from the MAT object containing the same 53 sequences as in Figure S1. The tree is rooted as is stored in the MAT object, i.e. at the reference sequence (not shown). The branch lengths are in number of parsimony steps, and the query sequence is represented by a bold black label.

Our objective here is to show an advantage of incorporating partially ambiguous sites into a typical analysis, and not a competitive comparison with UShER. It's possible, for instance, that one of the methods didn't select a given sequence due to the predetermined small neighbourhood size. Since UShER works with user-defined trees, this experiment suggests that perhaps one could use a distance-based dendrogram using uvaia-derived distances as input to it. Another approach would be to use UShER to define an initial set of neighbours, given its broad usage and continuous update of MAT objects for SARS-CoV-2 and scalability (given an existing MAT, UShER is much faster than uvaia), followed by a more detailed neighbourhood analysis with uvaia. It's worth keeping in mind that currently for SARS-CoV-2 the efforts to keep up-to-date phylogenetic trees make it more efficient to use e.g. the UShER ecosystem than to start from scratch, which should be considered in parallel to the novelties presented by uvaia.

# Difference between SNP distances and uvaia-based mismatches

In the main text, Figure 2 shows that the number of ACGT mismatches according to uvaia is usually higher than the SNP distance as inferred by snp-dists. In Figure S3 we display histograms of this difference for all pairwise comparisons, showing that both the number of "text matches" and "ACGT matches" can detect differences oblivious to a distance which excludes partially ambiguous sites. The figure furthermore shows that a distance based on the number of partial matches from uvaia is the more similar measure to the SNP distance. The exceptions are generally cases where there is a comparison between an unambiguous site and an incompatible ambiguous one, as for example between T in one sequence and R in the other (R is compatible with A and G, but not T). In these cases the distance based on partial matches will be higher than the SNP distance (which excludes such sites from the comparison), and in our small data set it occurred once at every 3,000 comparisons.

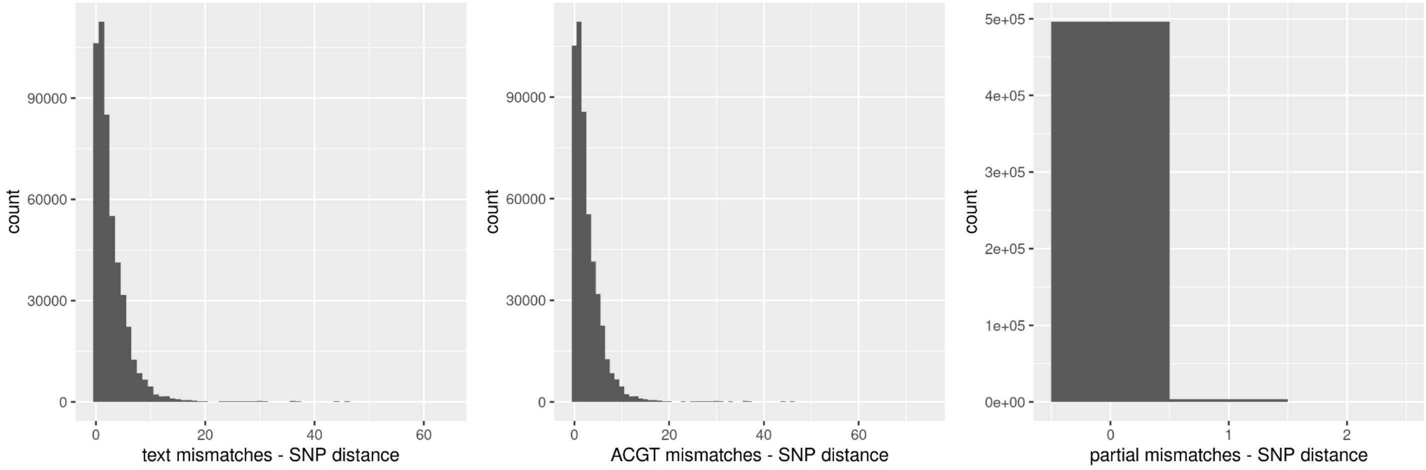


Figure S3. Histogram of the difference in mismatches between uvaia and snp-dists. While their difference is quite evident when we compare ACGT-only or text mismatches, the number of partial mismatches is almost identical to the SNP distance, differing at most by one in approx 3,000 pairs, out of the 499,500 comparisons.

# Time and memory requirements

To test the performance of uvaia we ran it using subsamples of the COG-UK unmasked alignment data set (archival copy available at https://webarchive.nationalarchives.gov.uk/ukgwa/20230507102210/https://www.cogconsortium.uk/priority-areas/data-linkage-analysis/public-data-analysis/ as of 2023.07.25). We used both a "large" machine (48 AMD cores with 248GB RAM) and a "small" one (8 AMD cores and 32GB of RAM memory) on the tests, where we

used default uvaia parameters and all available cores. The reference data set alignments (i.e. the larger set of sequences for which neighbours to each query sequence must be found) are always compressed with XZ. It is worth keeping in mind that the whole COG-UK data set used in this simulation study comprises almost 3 million sequences and takes 116MB when compressed with XZ.

The first benchmark is shown in Figure S4, where we fix the number of query sequences and vary the number of reference ones using the small machine. We see that the execution time is linear to the number of sequences in the reference database, while the peak memory usage does not change much, and is well below the small node capacity. For each combination of data sets, both the execution time and the maximum memory usage were recorded from the same uvaia run. The same behaviour was observed with the large machine, with shorter execution times as expected (result not shown). In Figure S5 we have a similar analysis where we fixed the reference data sets and varied the number of query sequences in the compressed alignment file. The memory usage is bound to reasonable values (way below 1GB) while the execution time increases a bit higher than linearly with the number of query sequences, due to the increased sequence diversity for larger query data sets (hampering search optimisations).

The pool size (i.e. how many reference sequences are loaded at once into memory) is determined automatically by uvaia in this case based on the number of cores available, and will influence the peak memory usage —influenced as well by the XZ decompression algorithm, by the query data set size and to a smaller degree by the number of neighbours to keep track of, and by the overall server load.

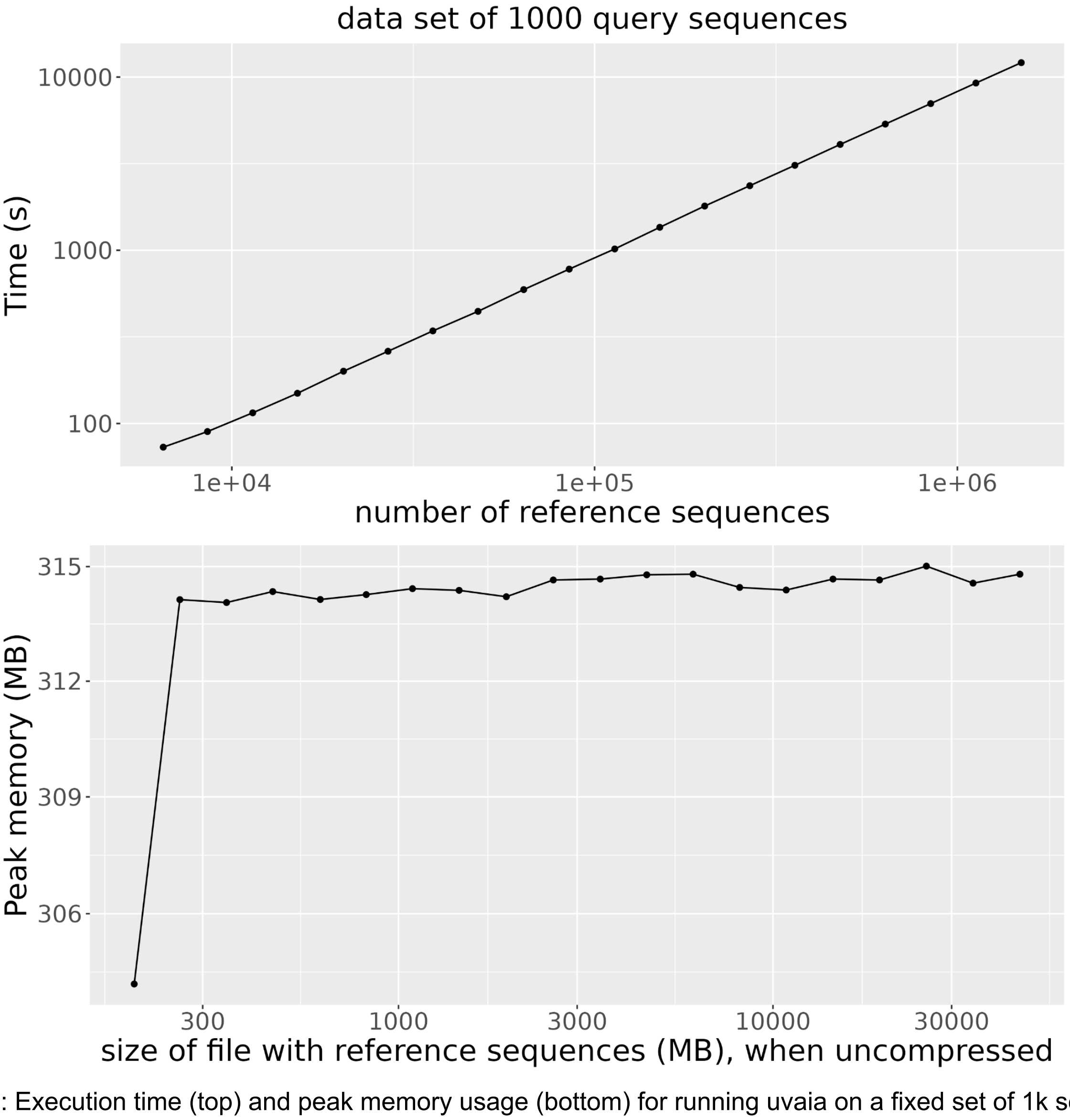


Figure S4: Execution time (top) and peak memory usage (bottom) for running uvaia on a fixed set of 1k sequences against a variable reference data set size on a small node. The X values on each panel correspond to each other, from a reference genome data set of 6471 SARS-CoV-2 genomes (200MB uncompressed) to a data set of approximately 1.5M

sequences (45GB uncompressed). The reference database files input to uvaia were always compressed (smaller than 100MB). Both axes are in log scale.

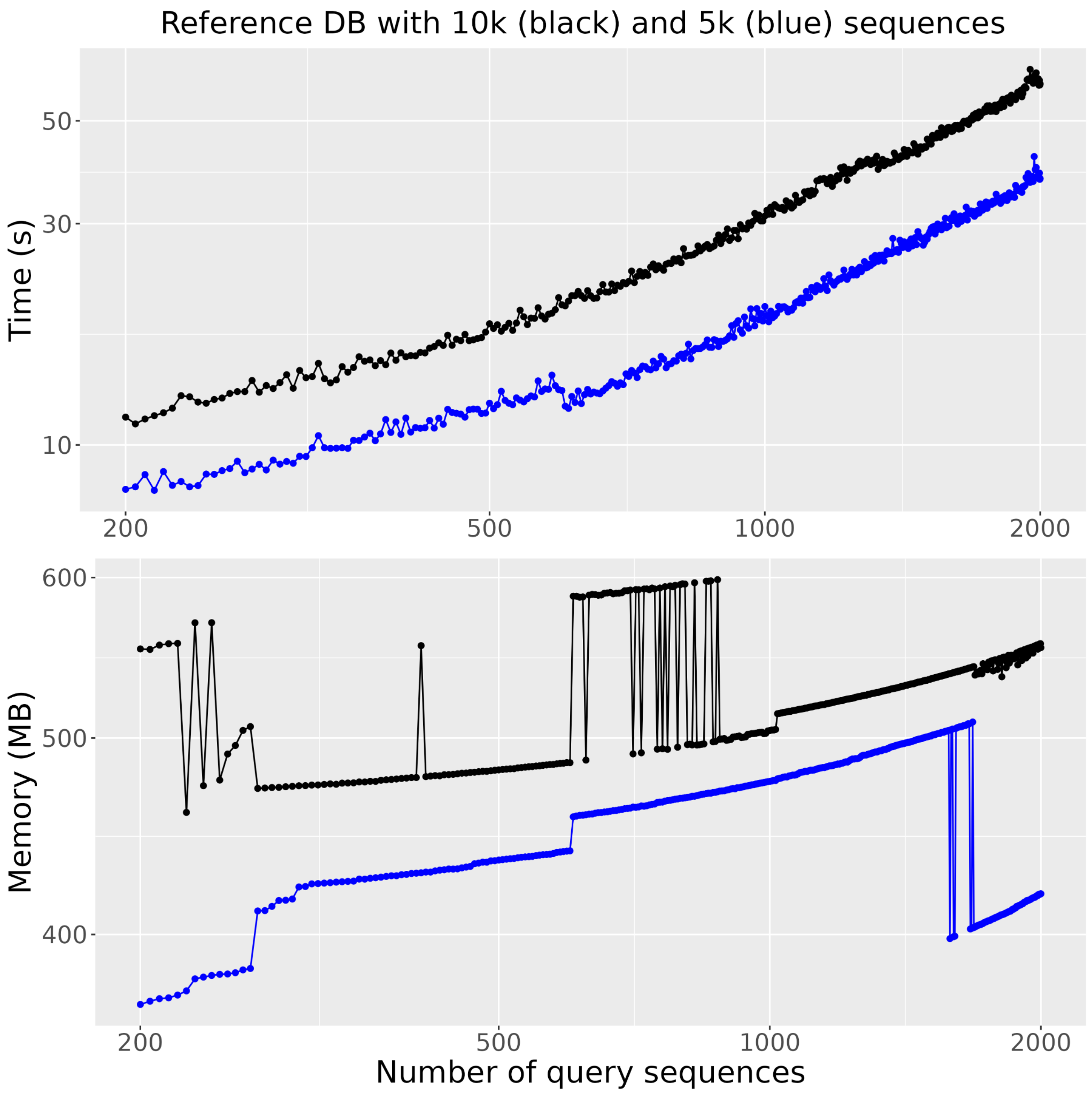


Figure S5: Execution time (top) and peak memory usage (bottom) for running uvaia on a fixed set of 5,000 (blue) or 10,000 (black) reference genomes on a large node. Both query and reference alignments were compressed with XZ, which may affect the peak memory use. Both axes are in log scale.

We also compared the execution time of uvaia with that of snp-dists, using 8 or 32 threads of the large machine for both programs. We varied the number of samples randomly, as before, but using the same random sample for both the query and reference sets for uvaia, since snp-dists requires a single alignment file. Here the alignment was not compressed to remove some execution overhead, although the output files generated by uvaia are still compressed with XZ. The results are shown in figure S6, where we see that snp-dists is faster than uvaia in particular for small data sets. This is because uvaia assumes that the number of reference sequences is potentially larger than the available memory, incurring in execution overheads and all query sequences being loaded into memory at once. The peak memory usage of snp-dists was also smaller than the one from uvaia, specially for a small number of sequences (results not shown).

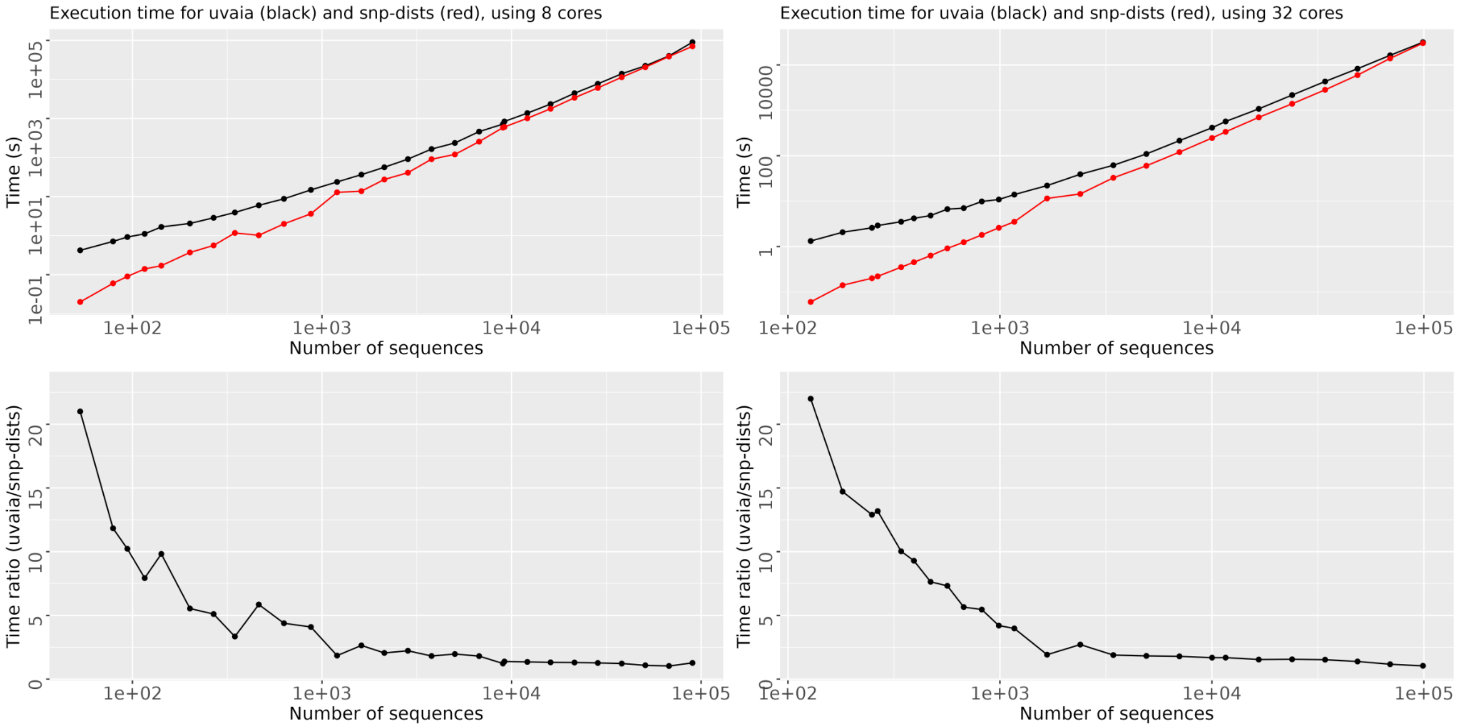


Figure S6: Comparison between the execution times of uvaia (black lines) and snp-dists (red lines) for a given same data set, with number of sequences varying from 53 and 99005, for 8 (left column) or 32 (right column) cores. The same alignment file is used as the query and reference data sets for uvaia, in order to be comparable with snp-dists. Both axes are in log scale. The top panel shows the timings for the individual tools, while the bottom panel shows the ratio between the timings per data set. The left panels show runs using 8 cores (for both snp-dists and uvaia) while the right panels show results using 32 cores.

# References


Burgess, R., & Yang, Z. (2008). Estimation of hominoid ancestral population sizes under bayesian coalescent models incorporating mutation rate variation and sequencing errors. *Molecular Biology and Evolution*, *25*(9), 1979–1994.

De Maio, N., Schlotterer, C., & Kosiol, C. (2013). Linking Great Apes Genome Evolution across Time Scales Using Polymorphism-Aware Phylogenetic Models. *Molecular Biology and Evolution*, *30*(10), 2249–2262.

Felsenstein, J. (2003). *Inferring Phylogenies*. Sinauer.

Hasegawa, M., Kishino, H., & Yano, T. (1985). Dating of the human-ape splitting by a molecular clock of mitochondrial DNA. *Journal of Molecular Evolution*, *22*(2), 160–174.

Joly, S., Bryant, D., & Lockhart, P. J. (2015). Flexible methods for estimating genetic distances from single nucleotide polymorphisms. *Methods in Ecology and Evolution / British Ecological Society*, *6*(8), 938–948.

Kozlov, A. M., Alves, J. M., Stamatakis, A., & Posada, D. (2020). *CellPhy: accurate and fast probabilistic inference of single-cell phylogenies from scDNA-seq data* (p. 2020.07.31.230292). https://doi.org/10.1186/s13059-021-02583-w

Kozlov, A. M., Darriba, D., Flouri, T., Morel, B., & Stamatakis, A. (2019). RAxML-NG: a fast, scalable and user-friendly tool for maximum likelihood phylogenetic inference. *Bioinformatics* , *35*(21), 4453–4455.

Potts, A. J., Hedderson, T. A., & Grimm, G. W. (2014). Constructing phylogenies in the presence of intra-individual site polymorphisms (2ISPs) with a focus on the nuclear ribosomal cistron. *Systematic Biology*, *63*(1), 1–16.

Scheunert, A., & Heubl, G. (2017). Against all odds: reconstructing the evolutionary history of Scrophularia (Scrophulariaceae) despite high levels of incongruence and reticulate evolution. *Organisms, Diversity & Evolution*, *17*(2), 323–349.

Turakhia, Y., Thornlow, B., Hinrichs, A. S., De Maio, N., Gozashti, L., Lanfear, R., Haussler, D., & Corbett-Detig, R. (2021). Ultrafast Sample placement on Existing tRees (UShER) enables real-time phylogenetics for the SARS-CoV-2 pandemic. *Nature Genetics*, 1–8.

Yang, Z. (1994). Maximum likelihood phylogenetic estimation from DNA sequences with variable rates over sites: approximate methods. *Journal of Molecular Evolution*, *39*(3), 306–314.

Yang, Z. (2014). *Molecular Evolution: A Statistical Approach*. Oxford University Press.

Ye, C., Thornlow, B., Hinrichs, A. S., Torvi, D., Lanfear, R., Corbett-Detig, R., & Turakhia, Y. (2022). matOptimize: A parallel tree optimization method enables online phylogenetics for SARS-CoV-2. In *bioRxiv* (p. 2022.01.12.475688). https://doi.org/10.1101/2022.01.12.475688

Zhao, L., Nielsen, R., & Korneliussen, T. S. (2022). distAngsd: Fast and accurate inference of genetic distances for Next Generation Sequencing data. *Molecular Biology and Evolution*. https://doi.org/10.1093/molbev/msac119
